# Chicago Medical Society

**Published:** 1879-03

**Authors:** 


### Society Reports.

### ARTICLE X.

### CHICAGO MEDICAL SOCIETY.

Meeting Feb. 3d, 1879, Dr. Hamill in the chair.

DR. F. C. HOTZ read an essay entitled: "Observations on Defects of Sight, induced by Exposure to Excessive Heat." In the latter part of last year a number of persons came under his care with defects of sight, which they noticed sooner or later after the very hot days in July. Two of these patients were not exposed to the direct rays of the sun, but were prostrated by the heat while working in hot rooms. The other patients were overpowered by insolation. They became dizzy, fainted and some were unconscious for several hours. The direct effect of the heat was a violent headache, lasting from several days to several weeks. During this period of headache the patients (except two) did not observe any visual disturbances; but after the headache ceased, vision became more or less indistinct. The ophthalmoscope revealed marked changes in the fundus oculi, viz.: in five cases neuro-retinitis, in one case choroiditis, with subsequent detachment of the retina. A careful analysis of his observations lead the reader to the view that the immediate effect of the excessive heat in these cases, was a hyperæmia of the meninges, in some cases bordering on inflammation; this hyperæmic or irritative state was propagated through the sheaths of the optic nerve to the eyeball, where it induced the neuro-retinitis or choroiditis. In support of this view the essayist adduced Schwalbe's anatomical investigations into the connection of the lymph spaces of the optic nerve with the sub-arachnoidal space; and analogous clinical observations which proved the fact that an inflammatory process of the meninges has been directly propagated to the eyeballs by the described route.

It was also stated that even the latest text-books on diseases of the eye do not speak of sunstroke as an indirect cause of visual disturbances.

In the discussion which followed, Dr. Montgomery said that, like the reader, he could not but be surprised at the almost entire lack of any literature on the subject. He reported a case of hyperæmia of the fundus oculi coming on suddenly after exposure to heat. He mentioned also two cases of retinitis due to observing the eclipse of last summer without the protection to the eyes of smoked glasses.

Dr. Ingals read a paper comprising a series of questions suggested by Dr. Sawyer's case of partial inversion of the uterus, reported in our January number. These were made the special topic of discussion for the next meeting.

Dr. Starkweather gave an un-official account of the work done by the State Board of Health. He stated the entire number of certificates issued to graduates and midwives as 4,171, while to those exempted under the ten-year clause, 942 certificates had been issued. He also gave an account of the efforts now making to repeal the laws creating a Board of Health and regulating medical practice, and thus open the field to every irregularity, as formerly.

